# Narratives of Crisis: Female Saudi Students and the Covid-19 Pandemic

**DOI:** 10.1177/10541373211029079

**Published:** 2023-01

**Authors:** Carmen Winkel, Beverley McNally, Siddiqua Aamir

**Affiliations:** 1 Core Curriculum Department, College of Sciences & Human Studies, Prince Mohammad Bin Fahd University, Al-Khobar, Saudi Arabia; 2 Human Resources Department, College of Business Administration, Prince Mohammad Bin Fahd University, Al-Khobar, Saudi Arabia

**Keywords:** Covid-19, Saudi-Arabia, female students, anxiety, depression, online teaching, crisis, narrative analysis

## Abstract

This paper endeavors to portray the narratives of a group of female undergraduate students, in Saudi Arabia, studying during the Covid-19 pandemic. Data collection occurred in March 2021, at which time students had been in online classes for a year and had experienced several curfews as well as a total lockdown. Students answered open-ended questions and wrote a reflection about how they experienced the pandemic. The majority of the participants reported sleep disturbances, depression, anxiety, and social isolation, identifying their family as the most important source of support during this time. Others tried to cope using their own resources. The study highlighted the need for increased awareness among faculty, education leaders and policy-makers about the psychological effects of lockdown and social isolation. There is a requirement for more support services for those affected by anxiety, depression, and regular follow-up after the pandemic ends to explore the long-term consequences.

## Introduction

Crises such as natural disasters, fires, political upheavals, rapid environmental changes and pandemics are nothing new to the human race. The word crisis is defined as a disruption or serious threat within a specific context (Koselleck & Richter, 2006). The three main characteristics of a crisis are first, a level of high uncertainty, second a high level of threat and third, they are primarily unanticipated (Seeger & Sellnow, 2016). As Seeger and Sellnow (2016, p. 71) state;

Few social phenomena are as complex, multifaceted, and dynamic and carry as much potential impact as large-scale crisis and disasters. These events bring together psychological, social, economic, political, technological, and environmental factors within the context of high uncertainty, high risk, and severe harm.

The current Covid-19 outbreak has unleashed a global pandemic causing one of the biggest crises since the Second World War and fits all three of these criteria (Snowden, 2019). On January 30 a matter of a few weeks after the initial occurrence of the virus, the World Health Organization (WHO) declared the outbreak of the novel-coronavirus to be a public health emergency, the highest level of alarm possible (Spinelli & Pellino, 2020). Approximately, one year later, WHO statistics identified a 110 million confirmed cases and 2.8 million deaths (World-Health-Organization, 2021). This, in turn, means many have suffered the loss of close family members, friends and work colleagues (Miller, 2020, p. 562). One year after the outbreak, the situation remains extremely tense in many countries. One statement that can be safely made is that is that there is virtually no individual, community or society that is not impacted in some way.

Covid-19 research studies have raised concerns the responses and restrictions may have created profound social and psychological damage (Odriozola-González et al., 2020; Vindegaard & Benros, 2020). The lockdown provisions have separated families. Young children separated from one or both parents, or elderly people from close family and support networks. Suddenly, habits and structures of everyday life were interrupted, and social contacts were hardly or not at all possible (Son et al., 2020). While, studies indicate the medical impact of Covid-19 virus may be less severe on children and young people, there is evidence to suggest long-term consequences could include negative impacts on their physical and mental health (Fegert et al., 2020). Moreover, their education and social life is predicted to suffer adverse consequences (Agbing et al., 2020, p. 3).

Young people had to disrupt their education overnight, switching from classroom based to online instruction (Aristovnik et al., 2020). School and university closures are still in place more than 12 months later. Studies reveal students have suffered social isolation, physical restrictions and drastic changes in their living conditions. Furthermore, in several countries students have been forced to leave the university campus. Thus, causing financial pressures, fear for their own health and that of their families being cited as the greatest stressors (Cao et al., 2020). Some have had difficulty in completing practical course work or carrying out research that involves contact with others. Consequently, the adverse economic and academic impact of the pandemic has and continues to cause distress for many students (Gonzalez et al., 2020). Thus, there have been calls for the development of tools and support systems specifically designed to support students’ educational and psychological well-being (Bourion-Bédès et al., 2021; Son et al., 2020; Tang et al., 2020; Ye et al., 2020).

In Saudi Arabia, universities closed on March 16, 2020, for face-to-face teaching, with all classes delivered online the next day. At the time of writing this paper, - some 14 months later, on the instructions of the Ministry of Education, all educational institutions remain closed. Schools and universities are not expected to open until the Fall of 2021. In addition to school and university closures, other far-reaching steps were taken in the Kingdom. Hard lockdowns and curfews imposed in the first half of 2020 included a 24-hour lockdown during Eid al Fitr 2020 at the end of Ramadan, both domestic and international air travel severely restricted. The latter stranding many citizens outside the country. Further precautionary measures included social distancing, the closure of restaurants, coffee shops, malls and all entertainment facilities (Bin-Dhim et al., 2021). While international air travel has been possible for short periods again, on March 15^th^ 2021 all international flights from and to Saudi Arabia were suspended. The ban not expected to be lifted until May 17 2021. The ban on praying in mosques, Umrah pilgrimages to Mecca, and the closure of Hajj to foreign pilgrims were among the more severe strategies taken to prevent the spread of the virus (Alshammari et al., 2020). With these tactics, Saudi Arabia has so far succeeded in keeping the number of infections and deaths relatively low compared to other countries. Data collection for our project took place in March 2021. At that time, the number of newly infected people were at 300-400 cases daily. On February 4, a 10-day closure of all restaurants and entertainment facilities was implemented. This was then extended when the rate of new daily infections suddenly rose to 1,000 plus (Saudi gazette, 2021).

As the first-year anniversary loomed the authors began to notice an increase in students speaking negatively about the restrictions they were facing. Students expressed frustration with online classes and lockdowns, mentioned disruptions in their sleeping and eating habits and some voiced concerns about their mental and physical health. These concerns acted as the motivators to commence an investigation aimed at identifying the how these students are managing both personal and academic issues during the Covid-19 crisis. Consequently, this study focuses on the following questions:

- How students experienced the lockdown and the pandemic
- How they cope with the pandemic
- What narratives were used when reflecting about the pandemic

The structure of the paper is as follows: first, introducing the study and the area of focus. The subsequent section provides a discussion of the narrative methodology, followed by the results of the study, then a presentation of the recommendations, limitations and conclusions.

## Methodology

A narrative is a story. With stories, humans give experiences a shape, they place characters in time and space and explain what has occurred (Bamberg, 2012). Telling and sharing stories, is an important part of human experience and provides meaning and understanding (Seeger & Sellnow, 2016). Abrams (2016, p. 109) defined a narrative as follows:

Narrative at its most basic level contains characters, a plot and a chronology. It is usually a communication about a life event and might take the form of any of a number of genres: a fairytale, a memory story, a speech, an anecdote, a folk- tale or an everyday speech act.

Traditionally, narrative studies have utilized in-depth interviews, biographical and autobiographical material (Atkinson & Delamont, 2006). However, the narrative method has progressed to include written data, chiefly, diaries, logs, letters or autobiographies (Elliot, 1997; McAlpine, 2016). In recent years many new sources of narrative data have been explored by social scientists like archival data, stories posted on the internet and social media and visual artefacts such as photography, videos and graphic novels (Murray, 2018). No matter the form, it is recognized that the use of stories as cultural and social objects, is a central element of human communication. Narrative analysis is concerned with the stories we share, how these stories are being constructed and how they help make sense of everyday events (Bamberg, 2012).

### Sampling Strategies

Participants were recruited from the female student population of a Saudi Arabian university. The sampling process was convenience sampling – the researchers had the ease of access to the students participants via email and on-line communication. Students were also asked to send the survey link to their peers if they were interested. The sample characteristics are outlined in Table 1. All participants were undergraduate students at the time of the survey. Participation for this study was voluntary and without compensation. Data for this study was collected in March 2021.

**Table 1.** Participants of the Study (N= 421).

Gender	Female	100%
Age	Younger than 20	47.4%
	Between 20–24	48.7%
	Older than 24	3.9%
Academic year	Freshman	58.7
	Sophomore	23.2
	Junior	12.9
	Senior	5.2
Major	Interior design	10.3
	HR	8.9
	Architecture	9.2
	Graphic design	3.4
	Electrical engineering	4.3
	Mechanical engineering	2.3
	Civil engineering	2.3
	Information technology	3.4
	Computer science	5.2
	Computer engineering	5.2
	Software engineering	9.5
	Accounting	2
	Finance	19.2
	Law	8.9
	MIS	6

## Data Collection and Analysis

For this study, we used written reflections of the participants that were collected with an on-line survey using Google forms. The first part of the survey was designed to establish demographic information (e.g. age, year of college, major) see Table 1. The second part was designed to elicit student reflections. The students were provided with basic guidelines to aid in their responses. The instructions recommended the reflection to be at least 400 words long and to focus on how the student experienced the Covid-Pandemic and its consequences. The prompt provided the students: *How did the Covid-19 pandemic and the lockdowns in Saudi Arabia affect you and your family. Please think about what effects, for example, online classes, social isolation, sleeping difficulties etc. have had on you.*

All reflections were anonymized, and permission was given by all participants for their use in a research project. All texts were given an ID number and extracted from Google forms. The first stage of the data analysis involved thematic analysis. The process following the six-steps recommended by Braun and Clarke (2006), familiarization with the data, the generation of initial codes, searching of themes, reviewing of themes, defining and naming of themes; and then the production of the report. During this process the reflections were analyzed, using an open coding process in order to identify themes (Braun & Clarke, 2006; Guest et al., 2011). In a second phase of the process we established those reflections that qualified as a narrative. For this purpose, we followed the criteria proposed by (Reese et al., 2011).

1. Context: author is giving orientation about time and place of the event.
2. Chronology: Author is giving a clear order of actions.
3. Theme: Author is writing about a clearly identifiable topic.

Applying these criteria, 325 of the 416 collected reflections were identified as narratives. These processes are now discussed in the following results section.

## Results

Themes were established from a cluster of several codes. Similar codes were reviewed, clustered and analyzed until eventually nine main themes emerged. These themes are outlined in Table 2.

**Table 2.** Distribution of Themes in Percent (421 Reflections).

Social isolation	26%
Struggle with online classes	23%
Loneliness/anxiety	18%
Depressive thoughts	10%
Family issues	7%
Disruption in sleeping patterns	6%
Concerns about family’s health	4%
Concerns about own health	2%

An example of how the thematic analysis provides a clear understanding of the participants’ struggles, fears and feelings is the naming of the theme *social isolation*. The theme *social isolation* took priority over the themes *struggle with online education* and *loneliness* and *anxiety.* The themes also showing that negative feelings and impacts outweighed the positive outcomes. Table 3 outlines the identified themes and examples of the students’ reflections. These reflections are reported in the original format, have not been edited, modified nor embellished

**Table 3.** Themes of the Reflections and Examples.

Themes	Example quotes
Loneliness/anxiety	Covid-19 had a huge effect on me and my family. I can't see my sister who lives far away, my social anxiety got worse and my friendships got decreased. My sister studies in the UK, so visiting her is not as easy as it was. Me and my family miss her more every day, and it hurts seeing her alone in a foreign country. My parents have chronic diseases, so me and my siblings always make sure that we aren't going to social gatherings like we used to. We would stay home most of our weekends and not visit any friends. That helped us prevent them from catching the virus, but it changed me so much. I always loved being alone and enjoy my own company from now and then however, now I cannot stand being around others without being afraid of being judged, my heartbeat would raise and I would feel really uncomfortable. That makes my energy drained and I would have that need of a gateway to run from these people and isolate myself again. This made my friendship circle tighten and I would not want to talk with anyone because I feel like there is not much to say and I would just feel awkward.
Depressive thoughts	Covid-19 has affected my lifestyle more than I have ever imagined. At First, the lockdown affected me in a very negative way, I started to get into a bubble of depression I could not do any task nor move out of bed, everything seemed boring and not nothing excites me anymore. Sadly, all I wanted to do was sleep and stay in bed. Furthermore, my brothers started to do some activities at home to entertain me. Thankfully, it helped me get motivated. But as soon as I knew that the online classes are still going on I fell into that depression mode. Online classes were the worst thing that I have gone through. In fact, online classes are not easy as everyone may think. It will affect our ability to socialize in the future because there is no direct interaction with people.
Disruption in sleeping patterns	The Covid-19 pandemic has been undoubtedly hard on everyone, but for me, I didn't mind the social isolation at first. However, as time went on, it gradually got worse and worse and so did my sleeping problems. To this day, I still struggle every night to sleep. I'm always sleepy and unfortunately sleep through most of my lectures. On the other hand, when I speak of my routine it would basically just be sleeping, eating, and attending online classes then spend some time with my family. My sleeping schedule is sadly ironic, because it's not even a schedule. I literally have 3 hours of sleep, then hop on to finishing work then sleep 2 hours wake up. I wouldn't call it a schedule, I'd rather call it free-styling
Struggle with online classes	Even I knew new friends from social media which is made me far away from social isolation. On other hand, online classes, till now I can't take it seriously, and I can't focus in the classes, I try my best to not get distracted. Also, there's a positive thing in online classes the way to university is too long so easily in online classes I can attend from my home.
Social isolation	This leads me to the topic of social isolation, where the support of your friends was no longer available unless it's through a screen which is completely different. Although I was able to contact friends and family online, it was extremely hard for me to just not be anywhere near them which greatly affected me emotionally. Our daily gatherings with friends in school and weekly gatherings with family during the weekend was suddenly stripped away from us due to the pandemic. Staying home with the same people and environment around you every day definitely starts affecting every person, whether they realizes it or not. You become unmotivated and unproductive due to being exposed to the same routine and environment with nothing exciting or motivating.
Family issues	My father is a businessman and has many different businesses one of them are his restaurants. He lost a lot of money because there weren't many people who would buy from it when the lockdown started. And as for my parents' relationship (…) so she does not spend much time with him, but once coronavirus lockdown started, they spent TOO MUCH time together, and that lead them to have a separation. My parents are now separated
Concerns about own health	In addition to that due to the fear of the virus my mental state forbids me to go out to public places because I’m too scared that I’ll catch the virus and die, which makes life a lot less fun.
Concerns about family’s health	However, the worst thing was when my grandmother was tested positive and was admitted to the ICU for 20 days. Thankfully she is better now. Since my sister works in the healthcare domain, we are always worried about her. This continuous fear has negatively affected our psychological health.
Concerns about the future	Finding a job, it is so hard to find jobs during the pandemic because most of the businesses and companies are falling and will not pay salaries to more people which is stressful for the new graduates and people with experiences and want to change the workplace like my brother.

**Table 4.** Coping Mechanism During the Lockdown.

Activity	Number
Family time	75
Hobbies	17
Chores	6
Exercise	8
TV/social media	44
Following government directives	56

**Table 5.** Types of Crisis Narratives.

Crisis narrative	Number
Blame	0
Hero	2
Memorial	8
Renewal	135
Victim	181
Total	326

### Coping Mechanisms

Participants’ responses identified a range of different coping mechanisms (Table 4). Among these were focussing more time and effort towards spending time with the family and focussing on their studies and on the future. Saudi Arabia is a collectivist society (Hofstede, 1983, 1991; House et al., 2002). Families in Saudi Arabia are usually much larger than in western countries and often comprise extended members of the family living together. Daily routine activities like cooking, cleaning, sleeping and eating were mentioned frequently. In addition to these activities the participants spoke of the importance of following government guidelines. These included activities such as social distancing, quarantine, mask wearing, and hand hygiene as the most important coping mechanisms. Leisure time activities such as watching TV, cooking, playing video games were also mentioned. Some participants also used the time to learn new languages or take up new hobbies.

## Discussion

Narrative expressions of a crisis are important vehicles for psychological healing and mental processing. These narratives aid the process of making meaning of the experience and helps expressing emotions and feelings (Seeger & Sellnow, 2016, p. 19). The five crisis narratives as identified by Seeger and Sellnow (2016) could be detected in the reflections we examined (Table 5). Among these, victim narratives were most frequently represented (181), followed by Renewal (135), Memorial (8) and Hero narratives (2), none were categorized as Blame.

From a narrative viewpoint, this tendency would cause individuals to express momentary thoughts or emotions without considering the broader context of the situation.

Hence, crisis narratives are not necessarily commentary about the political or social influences of a crisis, rather it provides a lens as to how people experience and deal with crisis on a personal level.

### Blame Narratives

Blame Narratives dealing with the question who is responsible for the crisis and call for punishment. They can deal with human or bureaucratic failure and ask how the crisis could have been avoided (Liu et al., 2020). Our participants did not use Blame narratives. The reasons proffered were, the proactive actions on part of the Saudi Government leading to the relatively low number of infections and Covid-related deaths.

### Hero Narratives

Hero Narratives refer to protagonists who are prevailing during a crisis. Through charisma, personal strength, intelligence and skills a person can have a disproportional impact on a community or society. Only two of the participants were highlighting single people for their role in the pandemic, interestingly in both cases mothers and sisters:

My mother and sister are practical examples of women who are on the front-line to help in the response to this pandemic. My mother has increased parenting duties because all my 3 siblings are at home and require special attention. My siblings are too young to understand what is really going on at this time. They are not aware of the reason they are at home at this time that they are required to be in school. I tried explaining to them that there is a disease outbreak but they couldn't easily understand. They probably think they have an extended holiday or something. However, my mother ensures that they wash their hands regularly and regulates their movement in and out of the house. They do not go out to play like they used to do, courtesy of my mother. She is a responsible woman who plays her parenting duties well to ensure our family is safe from this disease outbreaks.

### Memorial Narratives

Memorial Narratives create larger meanings about crisis (Liu et al., 2020). They celebrate human resilience, personal strength and healing. This type of narrative is very public and often used to make sense of a crisis for the community. Eight participants reflecting in this more in-depth way about the crisis:

I came to the realization that this is God’s will and we have to accept the way things are and know that everything happens for a reason. I’ve taken the time to reconsider my priorities and realize that God had bestowed many blessings in our life that are used in a daily basis but are still taken for granted and we got so used to it that we forget to thank God. I thank God, every day for the wellbeing of my family and having everything we need for the quarantine available. I also realized how our country is not like any other, they care about our health and welfare where they are spending billions just to ensure that we are safe which is amusing to many other countries knowing that their government are not supporting them, and their healthcare is collapsing. We are going through a tough time, but I know that this all will pass and become a memory and maybe this affliction had happened so that people can reconsider their priorities and realize that they have more than what they need.

When religious sentiments were used they expressed gratitude to God and to highlight the humbling effect the pandemic had on the person.

This pandemic changed life in one moment. We learned that we are the ones who plan, and God alone decides. It affected the State in general and society, and it affected the economy, education, and health centers, and it has almost exploded due to the large number of injuries and infections. Actually, I was not care about COVID-19 pandemic too much, I only listened to the government instructions. In 2020 I felt the blessings of God upon us and we were not known about all these blessings.

### Renewal Narratives

Renewal Narratives focusing on personal/administrative growth, healing and restoration (Liu et al., 2020). Renewal narratives often discuss a fresh start for an individual, community or society after the resolution of the crisis. A number of students expressed their gratitude towards the Government and King for the precautions that kept the country safe and the number of infections relatively low:

Now things are getting better with the Covid-19 deterioration because of the measures and procedures that the Government have taken and the vaccination that have started to be distributed.

Saudi Arabia does a lot of works to avoid this pandemic. Like there are a lot medicine and stuffs for many people. Also, we are thankful for our government. They keep doing their work for prevent us from this pandemic.

Several students highlighted positive effects of the lockdown and online education, especially the shared time with their families was described as one of the upsides of the pandemics:

The only thing that I can call as a good memory is when I used to sit outside with my family playing basketball, tennis, drinking coffee, watch movies. We became closer than ever. Sadly, not all my family members were there, my sister is working at the hospital, so she was working.

Participants described the crisis as a personal awakening and humbling experience that helped them appreciate things more:

Although the journey was stressful, but we learned lot form this pandemic in many aspects, we appreciated the time together and we became capable to face viruses which can be speeded worldwide like Covid-19.

Covid-19 pandemic affect my life in many ways. I knew the value of the simplest things like go out to get a cup of coffee! I learned how to communicate more with my family and appreciate simple everything.

### Victim Narratives

According to Seeger and Sellnow (2016) victim narratives are the most common crisis stories. Victim narratives deal with physical and emotional damage and suffering. These narratives personalize the harm caused by the crisis and focus on how a person suffered from a crisis that was not caused by the person themselves. In this study the majority of the students narrated the crisis using victim narratives. Focusing on the impact the crisis had on them personally, students described their fears after getting infected themselves and how isolation had a bearing on their physical and psychological well-being:

The biggest shock that happened to me was when I got COVID-19. It has shocked me because I was taking all the standard health precautions and even that I had it. I felt some symptoms such as; fever, cough, body pain, and I lose the sense of taste and smell. So, I isolated myself in my house for two weeks, they were the longest and most boring 14 days in my life.

Social isolation, missing seeing relatives, had a profound impact on participants and was a recurrent theme in their reflections:

The hardest part of all of this is being around your family but not that close, you want to see your friends but because you love them you will not see them, our grandparent always feel so bad and lonely because they do not use technology, so we miss them so much because we can’t call them by Facetime or another app.

Online instruction and the lack of interaction with students and instructors was described by many students as psychologically challenging, causing anxiety and depression. Contrary to teachers and university administrators perceiving it as welcome break.

Maintaining a healthy sleep schedule is difficult due to the fact that we are not moving anywhere in the morning, the only thing we are supposed to do is turn on the computer and log in. Furthermore, lockdown gave me anxiety, I do not feel comfortable going to public crowded places, I used to enjoy it but now I panic as soon as I go to packed place.

Students described episodes of anxiety and related mental and physical health issues.

Due to the fear of the virus my mental state forbids me to go out to public places because I'm too scared that I'll catch the virus and die, which makes life a lot less fun. Lastly Covid-19 made me stop going to the gym which affected me physically since I feel that I get easily tired and sleep all the time.

Staying home without regular social contacts outside their immediate family was experienced by many students as distressing which impacted them in various ways:

I never stayed at home for this long, this pandemic was a big challenge in my life. It made me an isolationist person, I even couldn’t study well as I should because I was in a bad mood, and of course my grade went down because of it. The days was repeated, we woke up, cooked dinner, watch movie, study, and then went to our bed again. There were nothing make you feel excited or want to live, you just waited until the end of the day to went to your pillow and share your sadness with it. I was waiting to feel alive again.

I feel really bad, because I don’t feel last day in high school, nor first day of university. Moreover, it’s really hard to keep focus without eye contact. I hope this is last month for covid, I hope we go back and enjoy our classes in the campus. And meet people.

It has been difficult to begin my university journey with minimum contact from fellow peers and students. Inevitably, this made me question how long this dilemma will last since I always believed that the people who you meet in this stage of your life are the key factors to making your college experience the most memorable—and experiencing it from behind a screen doesn't give you much to work with when it comes to socializing. Not to mention, sitting in the same chair the majority of your day to either attend classes, take tests, or work on assignments will make you feel overworked and instill fatigue within you. I often feel this way since I am in an environment that is not brain-stimulating and therefore I feel as though I am ceaselessly doing the same dull tasks. Because of this, I have noticed I have been chasing sleep to gain enough energy to do the next dull task that comes my way.

## Conclusion, Limitations and Recommendations

The objective of this study was to investigate how the participants experienced the pandemic, what coping mechanism they used and how they narrated the crisis. The narrative analysis brought the participants’ experience of the pandemic to the forefront. The evaluation of the informative narrative data provided an opportunity for the voices of the young women to be heard. In a patriarchal society this can be difficult to achieve. The data allowed insight into participants’ feelings, struggles and thoughts about the crisis. While experienced as an individual crisis the narratives impart a positive outlook for the future. As a renewal the participants expressed optimism about dealing with the crisis and emerging from it stronger.

Our data has shown that the crisis was experienced as a life-changing, disturbing and unsettling experience. Sleep disruptions, mood swings, and anxiety and depressive episodes were common. Feelings of loneliness, confusion and social isolation were also communicated along with the coping-mechanisms employed. Strengthening family ties through spending more time with them was mentioned as positive consequence of the curfews.

The data has shown that ‘going to classes’ was not a welcome relief. Participants reported problems with the on-line environment. Because of the instantaneous transition to online the students did experience technical difficulties. Consequently, there were technical difficulties including, stability with internet access, bandwidth issues with videos or sharing files or having the camera operational, server capacity, and the overall flexibility of how learning materials were presented. While these were coped with in the initial stages –the duration of the closure was optimistically set at 1-2 months. The students were aware that maybe the online platform was perhaps not the best alternative available. Participants queried if it was the most appropriate system for the longer term on-line environment. There was recognition that for many Faculty the on-line environment was just as new and unfamiliar as it was for the students. The university added an online module to the existing student learning management system as opposed to purchasing a specifically designed on-line platform. Additionally, the lack of interaction with fellow students and professors, (the informal interactions that happen on-campus) and the difficulty of concentrating on lessons were reported. While the lectures were the same length of time allocation as face to face teaching, they were reported as too long and requiring too much work. The participants stated that they had difficulty taking the classes seriously, hardly participating even if the professor asks questions and have considerable difficulty concentrating on the content. The lack of physical separation between home and university was deemed to be particularly disruptive.

The study has shown there is a need for more support services for those who are struggling with anxiety and depression. There are implications for teachers, administrators, and policy makers to pay more attention to the social, emotional, psychological, and physiological effects of lockdown. Psychological support via mobile counseling apps was available in Saudi Arabia during the lockdown but it is not clear how widely these services were used or known about (Hassounah et al., 2020). Confidential counseling involving social workers, video chats and telephone hotlines could be a way to assist students.

This study provides a sound baseline for future studies. For example, cross country comparisons. We suggest re-surveying students at one-year intervals to examine the long-term effects of the pandemic. In addition to psychological effects, we recommend investigating the social and educational impacts on students' academic performance. Future research may investigate the potential for a “Generation Corona” and whether this has negative consequences for future career and professional success. Our recommendations are supported by studies that highlight the need for mental health monitoring and assistance (Arslan et al., 2020; Bin-Dhim et al., 2021; Ye et al., 2020). Moreover, long lasting effects of crisis and disaster have been reported and need to be taken into consideration especially in the case of a worldwide pandemic (Bland et al., 1996; Fukasawa et al., 2020).

The limitations of the study arose from the hard-lockdown measures in Saudi Arabia. We were unable to meet with our participants in person and conduct interviews face to face. For this study only female students were approached. Further research including male students is required prior to generalizing the results to other groups. Future research comparing the experiences of female and male students would be desirable.
